# Riboflavin- and Hypericin-Mediated Antimicrobial Photodynamic Therapy as Alternative Treatments for Oral Candidiasis: A Systematic Review

**DOI:** 10.3390/pharmaceutics17010033

**Published:** 2024-12-28

**Authors:** Maciej Łopaciński, Jakub Fiegler-Rudol, Wojciech Niemczyk, Dariusz Skaba, Rafał Wiench

**Affiliations:** 1 Department of Periodontal Diseases and Oral Mucosa Diseases, Faculty of Medical Sciences in Zabrze, Medical University of Silesia, 40-055 Katowice, Poland; mlopacinski@sum.edu.pl (M.Ł.); niemczykwojciech00@gmail.com (W.N.); dskaba@sum.edu.pl (D.S.); rwiench@sum.edu.pl (R.W.); 2 Faculty of Medical Sciences in Zabrze, Medical University of Silesia, 40-055 Katowice, Poland

**Keywords:** aPDT, biofilm, Candida, denture stomatitis, diode laser, planktonic cells

## Abstract

**Background:** Oral candidiasis, predominantly caused by *Candida albicans*, presents significant challenges in treatment due to increasing antifungal resistance and biofilm formation. Antimicrobial photodynamic therapy (aPDT) using natural photosensitizers like riboflavin and hypericin offers a potential alternative to conventional antifungal therapies. **Material and Methods**: A systematic review was conducted to evaluate the efficacy of riboflavin- and hypericin-mediated aPDT in reducing Candida infections. The PRISMA framework guided the selection and analysis of 16 eligible studies published between 2014 and 2024. Data on light parameters, photosensitizer concentrations, and outcomes were extracted to assess antifungal effects. **Results**: Both riboflavin- and hypericin-mediated aPDT demonstrated significant antifungal activity, achieving substantial reductions in Candida biofilm and planktonic cell viability. Riboflavin activated by blue light and hypericin activated by yellow or orange light effectively targeted fluconazole-resistant Candida strains with minimal cytotoxicity to host tissues. However, complete biofilm eradication remained challenging, and variations in protocols highlighted the need for standardization. **Conclusions**: Riboflavin- and hypericin-mediated aPDT present promising, biocompatible alternatives for managing antifungal resistance in Candida infections. Further clinical trials and standardized protocols are essential to optimize outcomes and confirm efficacy in broader clinical settings.

## 1. Introduction

The oral cavity of humans is colonized by a diverse microbial community, predominantly composed of bacteria, with fungi constituting a smaller fraction [1]. Among these, *Candida* species are a natural part of the oral flora in healthy individuals, with *Candida albicans* being the most prevalent, accounting for 60–70% of cases, followed by *Candida tropicalis* and *Candida glabrata* [2]. While typically commensal, these yeasts can become pathogenic under specific conditions, leading to oral candidiasis [3]. *C. albicans* is the primary causative agent of oral candidiasis, responsible for up to 95% of cases [4]. Host factors such as xerostomia, smoking, oral prostheses, dental caries, diabetes, and cancer treatments can accelerate the disease process [5]. The immune response of the host mucosa plays a critical role in controlling *C. albicans*. Adaptive immune mechanisms, especially those mediated by Th1 and Th17 cellular responses, are essential for maintaining tissue homeostasis and combating fungal proliferation. Dendritic cells are pivotal in initiating these defenses by presenting antigens and producing cytokines [6,7]. However, systemic diseases or periods of immunosuppression may compromise these defenses, allowing *Candida* to exploit its virulence factors to establish and propagate infections [8]. The management of oral candidiasis typically involves topical or systemic antifungal agents, such as azoles and polyenes. However, the extensive use of these agents has led to the emergence of fluconazole-resistant *Candida* species [9]. Additionally, the formation of microbial biofilms provides a protective barrier, enhancing resistance to antifungal treatments and complicating disease management [10]. Given the rising prevalence of antifungal-resistant pathogens and the associated toxicity of conventional therapies, there is a pressing need for alternative strategies to control yeast infections [11]. Antimicrobial photodynamic therapy (aPDT) is emerging as a promising alternative. This technique involves the administration of a photosensitizing agent, which is activated by visible light at an appropriate wavelength to generate Reactive Oxygen Species (ROS) with antimicrobial effects [12]. The underlying mechanism of aPDT lies in its ability to induce oxidative stress, leading to microbial cell damage without significantly affecting host tissues. Importantly, aPDT has demonstrated efficacy against biofilm-associated infections and resistant strains, highlighting its clinical potential in managing recalcitrant fungal infections [13]. A critical aspect of aPDT is the selection of photosensitizers (PSs). In recent years, natural polyphenolic compounds have garnered interest due to their unique chemical structures, inherent antimicrobial properties, and favorable safety profiles. Curcumin, methylene blue, hypericin, and riboflavin have demonstrated strong antifungal activity in various studies [12,13,14]. Hypericin, derived from *Hypericum perforatum* (commonly known as St. John’s Wort), and riboflavin (vitamin B2) have shown promise against fluconazole-resistant strains of *Candida* [15,16,17,18,19]. Riboflavin, activated by blue light, produces ROS that induce oxidative damage to microbial cells, offering a natural and effective solution [20,21]. Given the increasing challenge of antifungal resistance and the need for innovative solutions, this review aims to systematically summarize the evidence supporting the use of polyphenolic natural products as photosensitizers in aPDT. By exploring their potential to counteract biofilm-related infections and resistant pathogens, this study seeks to establish a foundation for future clinical applications of aPDT in antifungal therapy.

## 2. Materials and Methods

### 2.1. Focused Question and Null Hypothesis

A systematic review was conducted following the PICO framework [22], as follows: In patients with *Candida* infections (Population), does treatment with riboflavin- or hypericin-mediated antimicrobial photodynamic therapy (Intervention), compared to blue light irradiation alone, the use of riboflavin or hypericin as photosensitizers alone, or other pharmacological treatments (Comparison), result in more effective eradication or reduction of *Candida* infections (Outcome)? The null hypothesis for our article stated that there is no significant difference in the eradication or elimination effectiveness of *Candida* strains when subjected to riboflavin- or hypericin-mediated antimicrobial photodynamic therapy compared to blue light irradiation alone, riboflavin or hypericin as photosensitizers alone, or other pharmacological treatments.

### 2.2. Search Strategy

**Figure 1:**
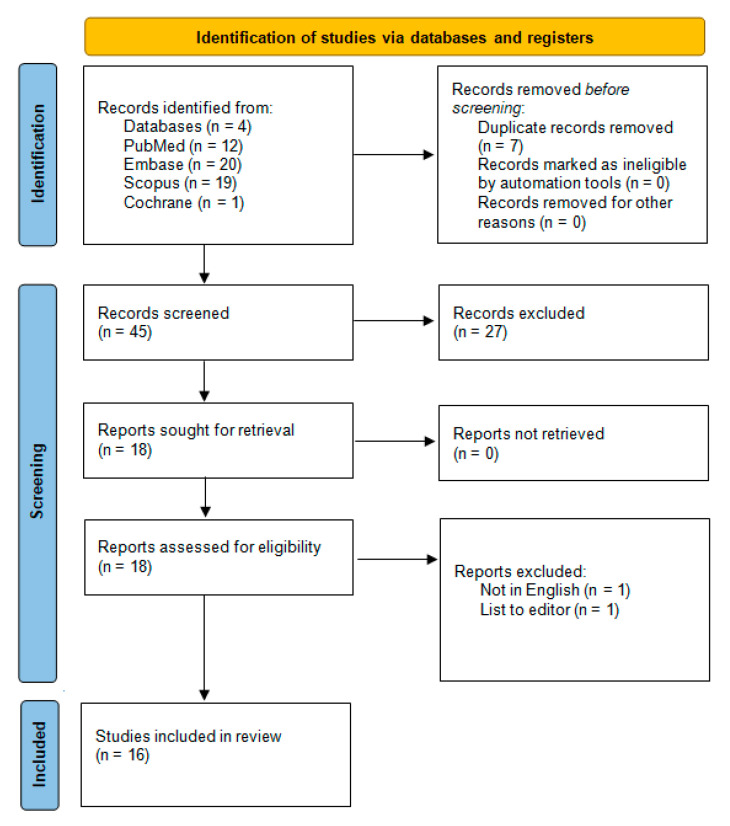
PRISMA 2020 flow diagram.

This review has been registered with PROSPERO under the ID CRD42024617727. The review was conducted following the Preferred Reporting Items for Systematic Reviews and Meta-Analyses (PRISMA) 2020 guidelines [23]. An electronic search was performed through PubMed/Medline, Embase, Scopus, and Cochrane Library databases (search phrases detailed in Figure 1). The databases were searched by three authors independently (M.Ł., J.F.-R. and R.W.) using the same search terms. Additional electronic filters were applied to include only articles published between 1 January 2014 and 3 December 2024 and restricted to publications in English. After initial screening, potential studies were selected based on titles and abstracts to determine if they met all inclusion criteria (Table 1). Two authors (J.F.-R. and R.W.) then conducted a full-text review of the selected studies to collate the desired data. Additionally, a snowball search was performed by examining the reference lists of articles deemed eligible for full-text review to identify further relevant studies. This research proposed that hypericin and riboflavin-mediated antimicrobial photodynamic therapy could serve as an effective approach for reducing Candida strains, potentially functioning as a complementary or alternative treatment for oral candidiasis compared to conventional pharmacological methods. Articles included in this review were selected based on specific inclusion and exclusion criteria.

### 2.3. Selection of Studies

During the study selection process for this systematic review, reviewers independently evaluated the titles and abstracts of the identified articles to minimize bias. Any disagreements regarding the eligibility of studies were resolved through collaborative discussions until a unanimous decision was achieved. This rigorous methodology, adhering to PRISMA guidelines, helped ensure that only the most relevant and high-quality studies were included in the analysis, thereby strengthening the review’s reliability and reproducibility [23].

### 2.4. Risk of Bias in Individual Studies

During the preliminary stage of study selection, reviewers independently examined titles and abstracts to minimize potential bias in the evaluation. Inter-reviewer agreement was measured using Cohen’s kappa statistic to ensure consistency in decision-making [24]. Disagreements concerning the inclusion or exclusion of studies were resolved through thorough discussions among the authors until a unanimous decision was achieved.

### 2.5. Quality Assessment

The quality of the included studies was independently assessed by two reviewers (J.F-R. and R.W.). The evaluation centered on critical elements of aPDT design, execution, and data analysis, with an emphasis on ensuring objectivity and result validation. The risk of bias was determined by assigning a score of 1 toeach “yes” response and 0 to each “no” response to a predefined set of evaluation criteria, as outlined below:

1. Was a specific concentration of riboflavin or hypericin as the photosensitizer indicated?
2. Was the origin or source of the photosensitizer (riboflavin or hypericin) provided?
3. Was the incubation time for the photosensitizer clearly stated?
4. Were detailed parameters of the light source (such as type, wavelength, output power, fluence, and power density) provided?
5. Was a power meter used in the study?
6. Was a negative control group included in the experimental design?
7. Were numerical results reported, including relevant statistics?
8. Was there no missing outcome data?
9. Was the study independent from its source of funding?

The data extracted from each study were analysed and categorized based on the total count of affirmative (“yes”) responses to the specified criteria. The level of bias was determined using the following scoring thresholds: high risk: 0–3; moderate risk: 4–6; and low risk: 7–9. Each study’s scores were compiled, and a corresponding level of bias risk—classified as low, moderate, or high—was determined in accordance with the guidelines specified in the Cochrane Handbook for Systematic Reviews of Interventions [25].

### 2.6. Risk of Bias Across Studies

The results of the quality assessment and risk of bias across the studies are presented in Section 3.3.

### 2.7. Data Extraction

After reaching a consensus on the selection of included articles, the two reviewers (J.F.-R. and R.W.) extracted data on various aspects, including the citation details (first author and publication year), type of study, Candida strains used, test and control groups, follow-up period, outcomes, type and parameters of the light source, concentration of riboflavin or hypericin, and the use of nanocarriers, additional substances, as well as incubation and irradiation times.

## 3. Results

### 3.1. Study Selection

Figure 1 illustrates the detailed research methodology per PRISMA guidelines [23]. The initial search yielded 52 articles, which were narrowed down to 45 after duplicate removal. Following the screening of titles and abstracts, 18 studies qualified for full-text evaluation. Of these, two were excluded. One study was excluded for not including *C. albicans*. One study was omitted as it was primarily written in a non-English language, with only the title and abstract available in English, and another was excluded for being a letter to the editor. Ultimately, 16 studies were included in the final review, all published within the last 10 years.

### 3.2. Quality Assessment Presentation

The risk of bias assessment for the 16 studies included after a full-text review is detailed in Table 2. Studies were required to score at least six points to be included in the analysis. Among these, all studies were identified as having a low risk of bias, with three achieving the maximum score of 9. None of the studies were rated as high or moderate risk.

### 3.3. Data Presentation

The extracted data from the 16 studies that met the eligibility criteria and were included in the review are summarized in Table 3, Table 4 and Table 5. These include an overview of the general study characteristics, the specifications of the light sources used, and the attributes of hypericin or riboflavin as a photosensitizer in aPDT protocols. The results of the quality assessment and risk of bias across the studies are presented in Table 3.

**Table 3:** The results of the quality assessment and risk of bias across the studies.

Reference Number	Author and Year	1	2	3	4	5	6	7	8	9	Sum	Risk
[26]	Agut et al. (2011)	1	1	1	1	0	1	1	1	1	8	Low
[27]	Alam et al. (2019)	1	1	1	1	1	1	1	1	1	9	Low
[28]	Alshehri et al. (2021)	1	1	1	1	1	1	1	1	1	9	Low
[29]	Arboleda et al. (2014)	1	1	1	1	0	1	1	1	1	8	Low
[30]	Ardakani et al. (2024)	1	1	1	1	1	1	1	1	1	8	Low
[31]	Bernala et al. (2011)	1	1	1	1	0	1	1	1	1	8	Low
[32]	Troichenko et al. (2021)	1	1	1	1	0	1	1	1	1	8	Low
[33]	Sakita et al. (2019)	1	1	1	1	0	1	1	1	1	8	Low
[34]	Sato et al. (2022)	1	1	1	1	0	1	1	1	1	8	Low
[35]	Tunccan et al. (2018)	1	1	1	1	0	1	1	1	1	8	Low
[36]	Rezusta et al. (2012)	1	1	1	1	0	1	1	1	1	8	Low
[37]	Morelato et al. (2022)	1	1	1	1	0	1	1	1	1	8	Low
[38]	Paz-Cristobal et al. (2014)	1	1	1	1	0	1	1	1	1	8	Low
[39]	Sato et al. (2023)	1	1	1	1	0	1	1	1	1	8	Low
[40]	Kashiwabuchi et al. (2013)	1	1	1	1	0	1	1	1	1	8	Low
[41]	Sousa et al. (2019)	1	1	1	1	1	1	1	1	1	9	Low

A comprehensive overview of the included studies is presented in Table 4.

**Table 4:** A general overview of the studies.

Reference Number	Author and Year	Country	Study Design
[26]	Agut et al. (2011)	Spain	In vitro study
[27]	Alam et al. (2019)	South Korea	In vitro and in vivo study
[28]	Alshehri et al. (2021)	Saudi Arabia	In vitro study
[29]	Arboleda et al. (2014)	USA	In vitro study
[30]	Ardakani et al. (2024)	Iran	In vitro study
[31]	Bernala et al. (2011)	Brazil	In vitro study
[32]	Troichenko et al. (2021)	Ukraine	In vitro study
[33]	Sakita et al. (2019)	Brazil	In vitro study
[34]	Sato et al. (2022)	Brazil	In vitro and in vivo study
[35]	Tunccan et al. (2018)	Turkey	In vitro study
[36]	Rezusta et al. (2012)	Spain	In vitro study
[37]	Morelato et al. (2022)	Croatia	In vitro study
[38]	Paz-Cristobal et al. (2014)	Spain	In vitro study
[39]	Sato et al. (2023)	Brazil and UK	In vitro and in vivo study
[40]	Kashiwabuchi et al. (2013)	Brazil and USA	In vitro study
[41]	Sousa et al. (2019)	Portugal	In vitro study

Main outcomes and details from each study are presented in Table 5.

**Table 5:** Main outcomes and details from each study.

Reference Number	Author and Year	Study Design	Species Evaluated	Study Groups	Outcomes
[26]	Agut et al. (2011)	In vitro study	*C. albicans*, *C. parapsilopsis*, *C. krusei*, *S. cerevisiae*	Hyp concentrations ranged from 0.625 μM to 320 μM +602 nm LED lamp at fluence levels of 0, 18, and 37 J/cm^2^.	Hyp was effective in aPDT for inactivating various yeast strains, including *C. albicans*, *Candida parapsilopsis*, *Saccharomyces cerevisiae*, and *C. krusei*. A 3-log reduction in fungal survival was achieved with lower concentrations of Hyp (0.625–40 µM), using a fluence of 37 J/cm^2^. For a 6-log reduction, higher concentrations of Hyp were required. The most sensitive strain was *C. albicans*. The aPDT treatment did not result in significant cytotoxicity in keratinocytes and dermal fibroblasts at the tested concentrations, suggesting the potential for therapeutic use with minimal side effects.
[27]	Alam et al. (2019)	In vitro and in vivo study	*C. albicans*, *S. aureus*, *MRSA*, *P. aeruginosa*	1. Control Group (no treatment) 2. Hyp only 3. Orange light only 4. Ampicillin only 5. Hyp + orange light (aPDT) 6. Hyp + Ampicillin + orange light 7. Positive Control Group (gentamicin or nystatin) 8. Gram-positive bacteria (*Staphylococcus aureus*, *MRSA*) 9. Gram-negative bacteria (*Pseudomonas aeruginosa*) 10. Fungal strains (*C. albicans*) 11. *C. elegans* in vivo model (treated vs. untreated pathogens)	aPDT using Hyp combined with orange light effectively inhibited *C. albicans*. A 4.8-log reduction in fungal growth was observed after treating *C. albicans* with 10 µM Hyp followed by orange light exposure for 1 h. This result demonstrated significant efficacy, comparable to the antifungal agent nystatin (20 µg/mL), confirming the potential of HY-PDT as an effective antifungal treatment.
[28]	Alshehri et al. (2021)	In vitro study	*C. albicans*	1. No decontamination 2. Nystatin suspension 3. RF 0.1% in darkness 4. Blue LED light only 5. RF-PDT with RF 0.1% + blue LED light	RF-PDT is highly effective in reducing *C. albicans* viability on acrylic denture surfaces. RF-PDT achieved the lowest *C. albicans* viability (41.1%) compared to nystatin (56.3%), riboflavin alone (81.7%), and no treatment (95.6%). SEM and CLSM analyses confirmed that RF-PDT-treated surfaces were almost completely free of *C. albicans*. Furthermore, RF-PDT caused no significant surface degradation or changes in mechanical properties of the acrylic resin, maintaining flexural strength and modulus. These results suggest that RF-PDT is a safe and effective antifungal method for denture disinfection.
[29]	Arboleda et al. (2014)	In vitro study	*C. albicans*, *F. solani*, *A*. *fumigatus*	1. Control—no treatment 2. 0.1% RB alone 3. 518 nm irradiation alone 4. RF-PDT (375 nm UV-A LED + riboflavin) 5. RB-PDT (518 nm green LED + rose bengal)	RB-PDT with 518 nm green light achieved the highest inhibition (95.6% ± 3%) of *C. albicans* growth compared to other treatment groups, including riboflavin-mediated aPDT with 375 nm UV-A light (35.3% ± 10%) and controls. RB alone and green light alone showed less inhibition (42.2% ± 3.7% and 35.6% ± 6.7%, respectively), while the untreated control exhibited 24.2% ± 2.6% inhibition. These findings indicate that RB-PDT is significantly more effective against *C. albicans* than riboflavin-mediated aPDT or individual components alone.
[30]	Ardakani et al. (2024)	In vitro study	*C. albicans*, *C. glabrata* (with *S. mutans* *S. sanguinis*)	1. Control 2. 0.2% chlorhexidine 3. 5.25% sodium hypochlorite 4. Curcumin 5. Riboflavin 6. Phycocyanin (each with and without LED)	aPDT using natural photosensitizers—curcumin, riboflavin, and phycocyanin—activated by LED light, was effective in reducing *C. albicans* in a multispecies biofilm model. aPDT with curcumin and LED light achieved the highest reduction in *C. albicans* biofilm, followed by riboflavin and phycocyanin. Significant decreases in biofilm mass and fungal cell viability were observed compared to untreated controls and groups treated with photosensitizers alone. The treatment showed promise for safe and effective antifungal disinfection of denture materials while maintaining their structural integrity.
[31]	Bernala et al. (2011)	In vitro study	*C. albicans*, *C. krusei*, *C. tropicalis*	1. Control group (*Candida* spp. without treatment) 2. PS group (Hyp only) 3. Light-only group (590 nm yellow LED light without Hyp) 4. aPDT group (Hyp + 590 nm yellow LED light) 5. Host cell control group (HEp-2 epithelial cells and McCoy fibroblasts treated with Hyp and light)	HY-PDT inactivation effectively reduced *C. albicans* viability under selective conditions. The optimal parameters included Hyp concentrations of 0.1–0.4 µg/mL, a short incubation time of 10 min, and irradiation with 590 nm yellow LED light at a dose of 6 J/cm^2^. These conditions resulted in significant photoinactivation of *C. albicans* without affecting mammalian fibroblast and epithelial cell viability, indicating high selectivity. The results suggest the potential of HY-PDT for treating oral candidiasis, particularly in immunocompromised patients, while minimizing damage to host tissues.
[32]	Troichenko et al. (2021)	In vitro study	*C. albicans* biofilm in ophthalmic model	1. Control group—no treatment 2. aPDT group—methylene blue + 630–670 nm laser 3. CXL group—riboflavin + 365 nm UV light 4. aPDT + CXL group—riboflavin, methylene blue, 365 nm UV light, and 630–670 nm laser 5. aPDT + CXL + antifungal group (fluconazole)	The study demonstrated that *C*. *albicans* is susceptible to aPDT with methylene blue (0.1%) and low-energy laser irradiation (630–670 nm), as well as CXL with RF (0.1%) and 365-nm ultraviolet light. Growth inhibition zones for aPDT alone ranged from 15 to 28 mm (mean 21.9 ± 4.7 mm), and for CXL alone, they ranged from 0 to 19 mm (mean 9.1 ± 9.6 mm). When combined (aPDT + CXL), inhibition zones ranged from 14 to 30 mm (mean 22.4 ± 7.27 mm). The most effective treatment was aPDT + CXL combined with fluconazole, producing the largest inhibition zones (36–38 mm, mean 36.9 ± 0.87 mm), 6.3 mm larger than the fluconazole control alone. These findings highlight the potential of combination treatments for enhanced antifungal effects against *C. albicans*.
[33]	Sakita et al. (2019)	In vitro study	*C. albicans*, *C. tropicalis*, *C. parapsilosis*, *C. glabrata*	1. Positive control 2. Light-only control 3. Photosensitizer-only control 4. aPDT + P123-Hyp at varying concentrations (0.25–32 µM) with specific light fluences (10.8–16.2 J/cm^2^) 5. Combination therapy with fluconazole (varied concentration) + P123-HY-PDT	P123-Hyp effectively inactivated *Candida albicans* through PDI. Using low Hyp concentrations (0.25–1.0 μmol/L) and light fluences of 10.8–16.2 J/cm^2^, the treatment achieved a significant reduction in *C. albicans* planktonic cells, with complete fungicidal effects at certain conditions. Additionally, P123-HY-PDT significantly inhibited biofilm formation, reducing *C. albicans* cell viability, metabolic activity, and total biomass by up to 87%. The therapy was synergistic with fluconazole, effectively overcoming fluconazole-resistant *C. albicans* strains by reducing the required drug concentrations up to eightfold. These findings support P123-Hyp-PDI as a promising strategy for treating *C. albicans* infections and preventing biofilm formation.
[34]	Sato et al. (2022)	In vitro and in vivo study	*C. albicans*	1. Negative control group: no treatment. 2. Infected positive control group: *C. albicans* inoculum without intervention. 3. Solvent control group—treated with 20% DMSO only. 4. Antifungal control group: Bio-vagin^®^ vaginal antifungal cream (contains benzoylmetronidazole, nystatin, and benzalkonium chloride). 5. Empty NLC-group. 6. Free Hyp-group: analyzed in the dark. 7. NLC-HYP-group: analyzed in the dark. 8. Free Hyp+ group: exposed to white LED light (113 J/cm^2^, 5 min). 9. NLC-HYP+ group: exposed to white LED light (113 J/cm^2^, 5 min).	NLC-HYP combined with aPDT effectively inhibited *Candida albicans* both in vitro and in vivo. In vitro, aPDT with NLC-HYP achieved a significant reduction in *C. albicans* planktonic viability, with a 90% reduction in fungal growth observed after a 5 min LED light irradiation. In vivo, using a murine vulvovaginal candidiasis model, NLC-HYP combined with aPDT significantly reduced fungal burden in vaginal lavages compared to untreated controls. Additionally, the treatment prevented the formation of pathogenic hyphae, promoting a predominance of yeast forms, which are less virulent. These results highlight the potential of NLC-HYP and aPDT as a promising therapeutic approach for treating *C. albicans* infections.
[35]	Tunccan et al. (2018)	In vitro study	*C. albicans*, *C. parapsilosis*	1. Red LED + MB group: biofilms treated with MB (25 µg/mL) and exposed to 660 nm red LED light. 2. Green LED + RB group: biofilms treated with RB (0.1%) and exposed to 518 nm green LED light. 3. RF + UV group: biofilms treated with RBF (0.1%) and exposed to 370 nm UV light. 4. Biofilm control: untreated biofilms grown on microplates and glass slides. 5. Negative control: biofilms treated with amphotericin B.	aPDT effectively reduced biofilm formation of *C. albicans* using a combination of red LED light (660 nm) and methylene blue as a photosensitizer. This treatment achieved a 45.4% reduction in biofilm formation, significantly outperforming other tested combinations such as riboflavin with UV-A and rose bengal with green LED light. In addition to biofilm reduction, aPDT markedly reduced the viability of *C. albicans* planktonic cells, with red LED + methylene blue treatment showing a 3-log10 reduction in fungal cell counts. These results highlight the potential of aPDT as a promising strategy to combat *C. albicans* biofilms, particularly in clinical settings involving catheter-associated infections.
[36]	Rezusta et al. (2012)	In vitro study	*C. albicans* strains resistant to azole	1. *Candida albicans* (ATCC and CECT strains): Hyp (0.625–640 µM), light fluences (18–37 J/cm^2^), suspensions (0.5, 4.0 McFarland). 2. *Candida parapsilosis* (ATCC 22019): Hyp (1.25–320 µM), light fluences (18–37 J/cm^2^), suspensions (0.5, 4.0 McFarland). 3. *Candida krusei* (ATCC 6258): Hyp (40–320 µM), light fluences (18–37 J/cm^2^), suspensions (0.5, 4.0 McFarland). 4. Dark controls. 5. Light-only controls. 6. Untreated controls.	HY-PDT effectively inactivated C. *albicans* under controlled conditions. A 3-log10 reduction in *C. albicans* viability was achieved at Hyp concentrations as low as 0.625 µM and light fluences of 18 J/cm^2^, while a 6-log10 reduction required higher Hyp concentrations (5 µM) and light fluences of 37 J/cm^2^. The treatment preserved the viability of human keratinocytes and fibroblasts when Hyp concentration and light fluence were maintained below these thresholds. These findings indicate that *C. albicans* is particularly susceptible to HY-PDT, making it a promising candidate for antifungal therapies with minimal damage to host cells.
[37]	Morelato et al. (2022)	In vitro study	*C. albicans*, *S. aureus*	1. Negative control 2. Positive control: Surface treated with a sterile cotton pellet soaked in 0.2% chlorhexidine-based solution for 60 s. 3. PDT1: Surface treated with 0.1% methylene blue dye as the photosensitizer, followed by 660 nm diode laser irradiation (124.34 W/cm^2^ power density, 1240 J/cm^2^ energy density) for 60 s. 4. PDT2: Surface treated with 0.1% riboflavin dye as the photosensitizer, followed by 445 nm diode laser irradiation (124.34 W/cm^2^ power density, 1.24 J/cm^2^ energy density) for 60 s.	RF combined with 445 nm diode laser light effectively reduced *Candida albicans* biofilm on titanium dental implants. Both aPDT with riboflavin and 445 nm blue light (PDT2) and aPDT with methylene blue and 660 nm red light (PDT1) achieved significant reductions in *C. albicans* CFUs compared to the untreated control group, with no statistically significant difference between the two aPDT protocols. These findings suggest that riboflavin and blue light offer an effective, aesthetically favorable alternative to methylene blue for managing fungal biofilms on dental implant surfaces.
[38]	Paz-Cristobal et al. (2014)	In vitro study	*C. albicans* (azole-resistant and azole-susceptible strains)	1. Negative control group 2. Hyp-group: different concentrations of Hyp (0.32–40 μmol/L), with light fluences of 18 or 37 J/cm^2^. 3. DMMB group: different concentrations of DMMB (0.32–40 μmol/L), with light fluences of 18 or 37 J/cm^2^. 4. ROS quencher groups: Sodium Azide: quenches singlet oxygen (¹O₂); Mannitol: quenches hydroxyl radicals (·OH); Catalase: quenches hydrogen peroxide (H₂O₂); Superoxide Dismutase: quenches superoxide anion radicals (O₂^−^).	aPDT using Hyp and DMMB effectively inactivated *Candida albicans* strains, including azole-resistant variants, in a light-dose and photosensitizer concentration-dependent manner. Hyp achieved a ≥3-log10 reduction at concentrations as low as 0.62 µmol/L for most strains, while DMMB required slightly higher concentrations (0.62–2.5 µmol/L). At higher fungal densities, Hyp required increased concentrations to achieve a 6-log10 reduction, while DMMB maintained efficacy with smaller concentration adjustments. ROS analysis revealed that Hyp primarily relied on hydrogen peroxide for its phototoxic effect, whereas DMMB depended on singlet oxygen. These results highlight aPDT as a promising strategy against azole-resistant *C. albicans*, with Hyp performing better at low fungal densities and DMMB offering advantages in high-density infections.
[39]	Sato et al. (2023)	In vitro and in vivo study	*C. albicans* with Gram-negative bacteria	1. PBS/Non-PDT (negative control) 2. Pure NLC/Non-PDT 3. Pure HG/Non-PDT 4. Free Hyp/Non-PDT 5. Free RB/Non-PDT (positive control) 6. PBS/PDT 7. Free Hyp/PDT 8. Free RB/PDT 9. Hy.NLC/PDT 10. Hy.NLC-HG/PDT	Hy.NLC-HG, combined with PDT, effectively reduced *Candida albicans* biofilm viability both in vitro and in vivo. In vitro, the Hy.NLC-HG/PDT treatment achieved a 1.2 log reduction in *C. albicans* viability, while other formulations, including free Hyp and rose bengal under PDT, showed reductions up to 1.9 log. In vivo, mice treated with Hy.NLC-HG/PDT displayed significantly lower fungal colony counts and a reduction in hyphae formation in vaginal fluids compared to controls. This treatment promoted a predominance of less virulent yeast forms of *C. albicans*, highlighting its potential as a non-invasive and effective approach for managing vulvovaginal candidiasis.
[40]	Kashiwabuchi et al. (2013)	In vitro study	*C. albicans* *F. solani*	1. Negative control group 2. UV-A light-only group: exposed to 365 nm UV-A light for 30 min without riboflavin. 3. Riboflavin-only group: fungal cells treated with 0.1% riboflavin solution without UV-A light exposure. 4. Dead cell control group 5. UV-A + riboflavin: fungal cells treated with riboflavin and exposed to 365 nm UV-A light for 30 min.	Combining RF with long-wave UV-A light (365 nm) had no significant antifungal effect on *C. albicans* in terms of cell viability reduction. While no notable cell death occurred, some phenotypic changes were observed, including a slight decrease in cell diameter and non-uniform biofilm growth in treated samples. These results suggest that the treatment may induce minor structural changes but is ineffective for directly inactivating *C. albicans*. Further optimization of dose and exposure time is needed to explore its potential as an antifungal treatment.
[41]	Sousa et al. (2019)	In vitro study	*C. albicans*	1. Tri-Py(+)-Me as photosensitizer: blood plasma samples treated with this porphyrinic compound at varying concentrations and subjected to photodynamic inactivation. 2. FORM as photosensitizer: a formulation based on a mixture of cationic porphyrins used for inactivation of *Candida albicans* in plasma and whole blood. 3. MB as reference photosensitizer: used for comparison in plasma and whole blood photodynamic therapy. 4. Light control group. 5. Dark control group. 6. DMSO control group: samples treated with the solvent used to dissolve porphyrinic photosensitizers to evaluate theirindependent effects.	The study found that PDT using porphyrinic photosensitizers, FORM and Tri-Py(+)-Me, effectively inactivated *Candida albicans* in blood plasma, achieving reductions of 1.7 log10 and 2.5 log10 in fungal viability at 10 µM concentrations, respectively, after 270 min of irradiation. Both photosensitizers outperformed MB, which only achieved a 0.5 log10 reduction under the same conditions. However, in whole blood, the reduction in *C. albicans* viability was modest, reaching only 0.7 log10 for both photosensitizers. Importantly, neither FORM nor Tri-Py(+)-Me caused significant hemolysis under isotonic conditions, supporting their potential for safe plasma disinfection applications.

PDT—Photodynamic Therapy, aPDT—Antimicrobial Photodynamic Therapy, Hyp—Hypericin, HY-PDT—Hypericin-mediated aPDT, LED—Light-Emitting Diode, CFU—Colony-Forming Units, SEM—Scanning Electron Microscopy, CLSM—Confocal Laser Scanning Microscopy, RB—Rose Bengal, RF-PDT—Riboflavin-mediated photodynamic therapy RB-PDT—rose bengal-mediated photodynamic therapy, UV-A—Ultraviolet A Light, DMMB—1,9-Dimethyl Methylene Blue, NLC—Nanostructured Lipid Carriers, Hy.NLC-HG—Hypericin-Loaded Nanostructured Lipid Carrier Hydrogel, MB—Methylene Blue, ROS—Reactive Oxygen Species, CXL—Collagen Cross-Linking, Tri-Py(+)-Me—Tri-Pyridyl Methyl, PC—Positive Control, NC—Negative Control, FLU—Fluconazole, P123-Hyp—Hypericin Encapsulated in P123 Micelles, RBF—Riboflavin, *C. albicans*—*Candida albicans*, *C. parapsilosis*—*Candida parapsilosis*, *C. krusei*—*Candida krusei*, *C. tropicalis*—*Candida tropicalis*, *C. glabrata*—*Candida glabrata*, *S. cerevisiae*—*Saccharomy cescerevisiae*, *S. aureus*—*Staphylococcus aureus*, MRSA—Methicillin-Resistant *Staphylococcus aureus*, *P. aeruginosa*—*Pseudomonas aeruginosa*, *F. solani*—*Fusarium solani*, *A. fumigatus*—*Aspergillus fumigatus*, SA—Sodium Azide, MAN—Mannitol, CAT—Catalase, SOD—Superoxide Dismutase, LC—Light Control, DC—Dark Control, HEp-2—Human Epithelial Type 2 Cells, MCF—McFarland Standard.

### 3.4. General Characteristics of the Included Studies

The general characteristics of the 16 studies that were included are shown in Table 4.

### 3.5. Main Study Outcomes

The reviewed studies collectively highlight the potential of aPDT using natural photosensitizers like hypericin or riboflavin as effective alternatives for managing *Candida* infections, especially in the context of rising antifungal resistance. Agut et al. demonstrated the broad-spectrum antifungal efficacy of hypericin, achieving a 3-log reduction in *C. albicans* and other yeast strains at low hypericin concentrations (0.625–40 μM) with a 37 J/cm^2^ fluence, with no cytotoxic effects on human keratinocytes or fibroblasts [26]. Alam et al. further validated hypericin’s effectiveness, showing significant inhibition of *C. albicans* and Gram-positive bacteria when activated by orange light, with combination therapy also enhancing outcomes for resistant strains like *P. aeruginosa* [27]. Alshehri et al. explored riboflavin-mediated photodynamic therapy (RF-PDT) on acrylic denture materials and found it highly effective for reducing *C. albicans* biofilm viability, outperforming other treatments and maintaining the mechanical properties of the materials [28]. Arboleda et al. demonstrated that rose bengal aPDT was more effective than RF-PDT at inhibiting *C*. *albicans*, while Ardakani et al. found that aPDT using riboflavin and other natural photosensitizers significantly reduced biofilm mass and *C. albicans* CFU counts in a multispecies biofilm model, showing promise for biofilm-related infections [29,30]. Bernala et al. highlighted hypericin fungicidal efficacy even at low concentrations, achieving selective *Candida* inactivation without harming host cells [31]. Sakita et al. enhanced hypericin’s efficacy by encapsulating it in micelles, leading to complete biofilm inhibition for multiple Candida species, with synergy observed when combined with fluconazole against resistant strains [33]. Sato et al. advanced these findings in vivo, showing that hypericin-loaded nanostructured lipid carriers combined with aPDT significantly reduced *C. albicans* colonies in a mouse model with minimal host tissue damage [39]. Troichenko et al. demonstrated the potential of combined aPDT and collagen cross-linking for inhibiting *C. albicans* biofilms in ophthalmic applications, while Morelato et al. found that riboflavin–PDT with blue LED and methylene blue–PDT with red LED both significantly reduced biofilm formation in peri-implantitis models [37]. Rezusta et al. showed that hypericin-mediated aPDT was effective against azole-resistant *C. albicans*, achieving a 3-log reduction at low fungal concentrations. These studies collectively underscore PDT’s potential as a minimally invasive, biocompatible, and effective treatment for fungal infections, particularly those associated with biofilms or resistant strains [36]. While many studies demonstrate the efficacy of aPDT, limitations such as incomplete biofilm eradication, variability in the reduction of fungal cell viability, and the potential for regrowth under suboptimal conditions must be acknowledged. Differences in light source parameters, such as wavelength and energy density, were observed to significantly influence outcomes, with some protocols achieving higher efficacy due to optimized settings. These findings highlight the importance of standardized methodologies to ensure reproducibility and to maximize the therapeutic potential of aPDT [28,29,30,31,32,33,34,35,36,37,38,39,40]. However, differences in experimental protocols, including photosensitizer concentrations, light parameters, and fungal strains, highlight the need for standardization. The lack of large-scale clinical trials is a limitation, as most evidence comes from in vitro or small animal studies. Future research should prioritize clinical validation and explore standardized aPDT protocols, innovative delivery systems like micelles or hydrogels, and combinations with antifungal drugs to optimize treatment outcomes and expand aPDT’s applicability across clinical settings. These findings suggest that aPDT, particularly with natural photosensitizers like hypericin and riboflavin, could offer a safe, sustainable, and effective alternative to traditional antifungal therapies [26,27,28,29,30,31,32,33,34,35,36,37,38,39,40,41,42].

### 3.6. Characteristics of Light Sources Used in aPDT

Table 6 outlines the physical parameters of the light sources used in studies that satisfied the inclusion criteria.

Table 7 summarises the concentration and incubation time of the photosenistisors used i the studies.

## 4. Discussion

### 4.1. Results in the Context of Other Evidence

This systematic review provides compelling evidence to reject our null hypothesis, which posited no significant difference in the efficacy of riboflavin- and hypericin-mediated aPDT compared to conventional antifungal treatments for managing *Candida* infections, demonstrating that aPDT is a promising alternative for reducing fungal load, particularly in cases where conventional antifungals face challenges due to resistance or biofilm formation. Both riboflavin and hypericin exhibited substantial antifungal activity against *Candida* spp. in vitro, with multiple studies achieving complete eradication of planktonic cells under optimized conditions [27,28,31,36], and were effective in reducing biofilm mass and viability, although biofilms remained more resistant than planktonic cells due to their structural complexity and extracellular matrix [32,33,35,36]. The versatility of riboflavin, activated by blue light, and hypericin, which responds to yellow or orange light, underscores their clinical potential, supported by safety profiles showing minimal cytotoxicity to human keratinocytes and fibroblasts, indicating their suitability for clinical applications without damaging host tissues [31,36,39]. Riboflavin- and hypericin-mediated aPDT effectively targeted fluconazole-resistant *Candida* strains, offering a promising strategy to address antifungal drug resistance, while their combination with conventional antifungal agents like fluconazole enhanced effects, particularly against biofilm-associated infections, suggesting a dual-action therapeutic approach [32,33,36]. Despite significant reductions in *Candida* spp. cell counts, complete biofilm eradication was not consistently achieved due to the biofilm’s protective extracellular matrix emphasizing the difficulty of treating biofilm-associated infections, and the findings remain limited by the predominance of in vitro studies and the variability in study designs, including differences in light parameters, photosensitizer concentrations, and incubation times, complicating direct comparisons and underscoring the need for standardized protocols [35,36,37]. Nonetheless, in vivo studies confirmed the effectiveness of hypericin-loaded hydrogels in reducing *Candida albicans* vaginal colonies and riboflavin-mediated aPDT in decreasing fungal loads on dental surfaces and prosthetic materials, with both photosensitizers identified as promising natural alternatives due to their biocompatibility, low toxicity, and sustainable use in aPDT though the scarcity of in vivo or clinical trials limits the generalizability of results, requiring future research with large-scale studies to establish standardized protocols and optimize outcomes [26,36]. These findings align with prior evidence emphasizing the potential of photodynamic therapy in overcoming antimicrobial resistance and managing biofilm-associated infections, underscoring its promise for clinical application in drug-resistant *Candida* infections and biofilm-associated conditions. Numerous studies have highlighted the promising role of photodynamic therapy in managing oral candidiasis, strongly supporting its potential as an effective alternative or adjunct to traditional antifungal treatments. For instance, Hu et al. evaluated the efficacy of PDT in comparison to conventional antifungal drugs and found it to be superior to nystatin in reducing *Candida* colonies [42]. Their findings also suggested that PDT might offer greater effectiveness than other antifungals, such as fluconazole and miconazole. Rodríguez-Cerdeira et al. similarly reported that PDT is a promising treatment option due to its broad antimicrobial spectrum and the ability to target drug-resistant strains of Candida, making it a viable alternative where traditional antifungal treatments might fail [43]. Supporting this, D’Amico et al. demonstrated that PDT significantly reduces *Candida albicans* biofilm without exerting cytotoxic effects on gingival cells. Their study compared various photosensitizers and reinforced PDT’s potential as both a primary and adjunctive therapy for oral candidiasis [44]. The mechanisms underlying PDT’s effectiveness were further explored by Kashef and Hamblin, who noted its ability to alter Candida cell permeability, ultimately leading to fungal death [45]. They also highlighted PDT’s wide antibacterial spectrum, short therapeutic course, and strong targeting capabilities, which make it particularly advantageous in antifungal treatment strategies. However, the application of PDT shows variability depending on the site of treatment. For example, Agut et al. observed that PDT was more effective in reducing *Candida* colonies on the palate compared to the denture area [26]. This difference is attributed to the porous and irregular surfaces of dentures, which facilitate microbial adhesion and recolonization. The limited action of PDT on denture surfaces compared to broader oral areas treated with systemic drugs further underscores this finding. Studies by Pérez-Laguna et al. and others have emphasized that mechanical cleaning of dentures is essential to achieve optimal outcomes, as PDT alone is insufficient to address fungal growth on such surfaces [46]. Comparative analyses have further clarified PDT’s position relative to other antifungaltreatments. For example, while PDT showed similar efficacy to fluconazole, miconazole was found to be more effective [47]. However, PDT’s ability to target drug-resistant Candida strains gives it a distinct advantage over azoles in specific cases. Furthermore, systemic antifungals like amphotericin B demonstrated less effectiveness in removing fungal colonies from denture surfaces, further reinforcing the critical role of regular denture cleaning in managing oral candidiasis [48]. Despite these advancements, recurrence rates of candidiasis remain a significant concern. Studies by Mima et al., Macial et al., and Schwingel et al. have reported high recurrence rates, primarily due to inadequate denture cleaning and Candida recolonization [49,50,51]. Nevertheless, the combination of PDT with traditional antifungal agents has shown promise in mitigating these challenges [42]. Notably, the synergistic effect of PDT and nystatin has been demonstrated to significantly reduce Candida colonies and lower recurrence rates. This is attributed to their complementary mechanisms of action, which enhance overall treatment efficacy [42]. Studies by Paz-Cristobal et al. and Agut et al. highlighted that combination therapy with PDT and nystatin is both effective and safe, with adverse reactions reported as mild and self-limiting [26,38]. Common side effects, such as nausea and burning tongue, were mostly observed in immunodeficient patients and did not pose significant risks [42,52,53]. Overall, PDT presents itself as a valuable adjunct to traditional antifungal therapies due to its broad spectrum, strong targeting capability, and short therapeutic course. Combining PDT with antifungal agents like nystatin is recommended for achieving improved clinical outcomes and reducing the recurrence of oral candidiasis [42]. These findings collectively underline the potential of PDT as an innovative and effective approach tothe management of this challenging condition [36,37,38,39,40,41,42].

### 4.2. Limitations of the Evidence

The primary limitation lies in the significant variability of protocols across studies. Light source parameters, including wavelength, energy density, and fluence, as well as photosensitizer concentrations and incubation times, differed widely, making it difficult to compare outcomes or establish standardized guidelines. Furthermore, while many studies reported efficacy against *Candida* spp., most were conducted in vitro, with limited in vivo evidence or clinical trials available to confirm these findings in human populations. Additionally, while aPDT was effective in reducing biofilm mass, complete eradication of biofilms was rarely achieved, highlighting a persistent challenge in biofilm-associated infections. Finally, the studies included in this review often relied on subjective outcome measures, such as CFU reduction and visual biofilm assessment, without integrating advanced diagnostic tools like imaging or molecular analyses to substantiate their results.

### 4.3. Limitations of the Review Process

The lack of homogeneity among the included studies led to a narrative synthesis of results. The variability in study designs, intervention protocols, and outcome measures may have introduced bias in evaluating the overall efficacy of riboflavin- and hypericin-mediated aPDT. Moreover, the significant differences in the parameters used across studies prevented the authors from applying the GRADE tool, and as such, heterogeneity makes it challenging to formulate clear recommendations. To address this, future research should focus on conducting well-designed randomized controlled trials to directly compare specific parameters and establish standardized protocols. Furthermore, the exclusion of non-English studies and gray literature may have restricted the scope of the review, potentially overlooking relevant data. These limitations highlight the need for more rigorous research in this field to enable systematic comparisons and quantitative analyses.

### 4.4. Implications for Practice, Policy, and Future Research

Riboflavin- and hypericin-mediated aPDT shows promise as a safe and effective alternative or adjunct to conventional antifungal therapies, particularly for managing drug-resistant or recurrent Candida infections. Clinicians should consider incorporating aPDT into treatment protocols for conditions such as denture stomatitis and other mucosal infections. However, for this to become routine practice, standardized treatment protocols—including consistent light parameters, photosensitizer concentrations, and application techniques—must be developed and validated. Policymakers and research funding agencies should prioritize support for large-scale, multicenter clinical trials to confirm the efficacy and safety of aPDT in diverse patient populations. Furthermore, future research should explore the synergistic potential of combining aPDT with existing antifungal drugs, particularly for biofilm-associated infections where monotherapies often fall short. Innovations such as nanocarrier systems and hydrogels could enhance the delivery and stability of photosensitizers, improving therapeutic efficacy. While the current evidence highlights the potential of aPDT in combating oral candidiasis and related infections, addressing the identified gaps is crucial for its integration into routine clinical practice.

Future research should prioritize the standardization of parameters to enable more reliable and meaningful comparisons across studies. Moreover, further investigation is needed to examine the variations in efficacy between different regions of the mouth, such as tooth versus palate applications, to deepen our understanding of site-specific outcomes.

## 5. Conclusions

This systematic review demonstrates the potential of riboflavin- and hypericin-mediated antimicrobial photodynamic therapy as promising, non-invasive alternatives for managing Candida infections, particularly in cases resistant to conventional antifungal therapies. Significant reductions in fungal viability and biofilm mass, coupled with minimal cytotoxic effects, highlight the clinical promise of these photosensitizers. Riboflavin and hypericin, activated by blue and yellow/orange light, respectively, offer versatility and efficacy, particularly against fluconazole-resistant strains. Additionally, the reviewed evidence indicates that aPDT can achieve outcomes comparable to traditional antifungal agents while reducing systemic side effects and potentially enhancing efficacy through synergistic combinations. However, the lack of standardization in study protocols, such as variations in light parameters and photosensitizer concentrations, alongside a scarcity of large-scale clinical trials, limits the applicability of these findings. To address these limitations, future research should prioritize large-scale, multicenter studies with consistent methodologies and explore optimal treatment parameters to enhance the reproducibility and effectiveness of aPDT. Furthermore, focusing on areas where aPDT could have the most immediate clinical impact, such as the treatment of antifungal-resistant Candida strains and biofilm-associated infections, would maximize its relevance and utility. These targeted applications address critical gaps in current antifungal therapies and could accelerate the integration of aPDT into clinical practice.

## Figures and Tables

**Table 1:** Search syntax used in the study.

Source	Search Term	Filters	Number of Results
PubMed/MEDLINE	(Candida [Title/Abstract] OR candidiasis oral [Title/Abstract] OR denture stomatitis [Title/Abstract]) AND (photodynamic therapy [Title/Abstract] OR aPDT [Title/Abstract] OR antimicrobial photodynamic therapy [Title/Abstract] OR photodynamic antimicrobial chemotherapy [Title/Abstract] OR PACT [Title/Abstract] OR photodynamic inactivation [Title/Abstract] OR PDI [Title/Abstract]) AND (riboflavin [Title/Abstract] OR hypericin [Title/Abstract])	English language Publicationyears: 2014–2024 Full text	12
Embase	(’candida’:ti,ab OR ’candidiasis oral’:ti,ab OR ’denture stomatitis’:ti,ab) AND (’photodynamic therapy’:ti,ab OR ’aPDT’:ti,ab OR ’antimicrobial photodynamic therapy’:ti,ab OR ’photodynamic antimicrobial chemotherapy’:ti,ab OR ’PACT’:ti,ab OR ’photodynamic inactivation’:ti,ab OR ’PDI’:ti,ab) AND (’riboflavin’:ti,ab OR ’hypericin’:ti,ab)	Publication years: 2014–2024 Controlled Clinical Trial Randomized Controlled Trial	20
Scopus	(TITLE-ABS (“Candida”) OR TITLE-ABS (“candidiasis oral”) OR TITLE-ABS (“denture stomatitis”)) AND (TITLE-ABS (“photodynamic therapy”) OR TITLE-ABS (“aPDT”) OR TITLE-ABS (“antimicrobial photodynamic therapy”) OR TITLE-ABS (“photodynamic antimicrobial chemotherapy”) OR TITLE-ABS (“PACT”) OR TITLE-ABS (“photodynamic inactivation”) OR TITLE-ABS (“PDI”)) AND (TITLE-ABS (“riboflavin”) OR TITLE-ABS (“hypericin”))	Article Publication years: 2014–2024	19
Cochrane	((mh “Candida” OR “candidiasis” OR “denture stomatitis”) AND (“Photodynamic Therapy” OR “antimicrobial photodynamic therapy” OR “PDI” OR “aPDT”) AND (“Riboflavin” OR “Hypericin”))	Publication years: 2014–2024	1

**Table 2:** Selection criteria for papers included in the systematic review.

Inclusion Criteria	Exclusion Criteria
Studies that assess Candida elimination through riboflavin-mediated aPDT or hypericin-mediated aPDT in both in vitro and animal models. In vitro animal studies involving strains from the Candida genus of yeasts. RCTs where riboflavin or hypericin was used as the primary photosensitizer in aPDT for Candida treatment. Studies evaluating synergistic effects of riboflavin or hypericin aPDT combined with other antifungal agents. Research with control groups that assess the effects of aPDT with riboflavin or hypericin against untreated controls or alternative therapies. Comparative studies that directly compare the efficacy of riboflavin- and hypericin-mediated aPDT with other antifungal treatments. Longitudinal studies or studies with follow-up periods to monitor the antifungal effects of riboflavin or hypericin aPDT.	“Gray literature” sources Study types: case reports or case series, letters to the editor, historical overviews, narrative or systematic reviews, books, documents, and other non-journal sources. Non-peer-reviewed sources Studies published in languages other than English. Duplicate publications or studies sharing the same ethical approval number. General medical applications unrelated to oral candidiasis. Studies lacking a control group or comparison group Use of aPDT not intended as a therapeutic method for Candida. Other photosensitizers than riboflavin or hypericin. Studies on non-Candida infections or studies that do not evaluate Candida strains. In vitro studies that do not simulate oral conditions relevant to Candida infections

aPDT: antimicrobial photodynamic therapy; RCT: randomized controlled trial.

**Table 6:** Light sources’ physical parameters of studies that fulfilled the eligibility criteria.

Reference Number	Author and Year	Light Source	Wavelength	Energy Density (Fluence) (J/cm^2^)	Power Output (Standardized) (mW/cm^2^)	Irradiation Time (s)	Spot Size/Fiber Surface Area (cm^2^)
[26]	Agut et al. (2011)	LED	602	0, 18, 37	Not specified	Not specified	Not specified
[27]	Alam et al. (2019)	Orange LED	590	Not specified	15 ± 2	3600–10,800	Not specified
[28]	Alshehri et al. (2021)	Blue LED	450	15	25	600	4.5
[29]	Arboleda et al. (2014)	Green and UV LED	375 and 518	5.4	2.2	Not stated	28.3
[30]	Ardakani et al. (2024)	Blue LED Red LED	410–490 630–640	60	1100 ± 200	60 20	0.5
[31]	Bernala et al. (2011)	Yellow LED diffusion table	590	6	2000 ± 400	600	Not specified
[32]	Troichenko et al. (2021)	UV and low-energy lasers	365	Not specified	10	600 (UV) 180 (laser)	Not specified
[33]	Sakita et al. (2019)	Nanocarriers with PDT	450 to 750	16.2	Not specified	Not specified	Not specified
[34]	Sato et al. (2022)	Hypericin-loaded LED system	532	Not specified	3.0	Not specified	Not specified
[35]	Tunccan et al. (2018)	LED and UV-A	LED (660 and 528) UV-A (370)	0.233	Not specified	300	Not specified
[36]	Rezusta et al. (2012)	LED	602 ± 10	18, 37	0.077 (assumed area: 10 cm^2^)	Not specified	Not specified
[37]	Morelato et al. (2022)	Diode laser	445	1.24	Not specified	60	Not specified
[38]	Paz-Cristobal et al. (2014)	LED	602 ± 10	37	124,340	Not specified	Not specified
[39]	Sato et al. (2023)	LED	525	113	10.3	900	Not specified
[40]	Kashiwabuchi et al. (2013)	UV-A (365 nm) with riboflavin	365	5.4	Not specified	1800	Not specified
[41]	Sousa et al. (2019)	PAR radiation, OSRAM 21 lamps of 18 W	380–700	Not specified	3.0	16200	Not specified

LED: Light-Emitting Diode; UV: Ultraviolet.

**Table 7:** Characteristics of PS used in studies meeting eligibility criteria.

Reference Number	Author and Year	Incubation Time (Minutes)	Concentration/s of PS Used
[26]	Agut et al. (2011)	Not specified	0.625 M hypericin 1.25 M hypericin 2.5 M hypericin 40 M hypericin
[27]	Alam et al. (2019)	30	0.1% hypericin
[28]	Alshehri et al. (2021)	10	0.1% riboflavin
[29]	Arboleda et al. (2014)	Not specified	0.1% riboflavin 0.1% rose bengal
[30]	Ardakani et al. (2024)	Not specified	Curcumin Riboflavin Phycocyanin
[31]	Bernala et al. (2011)	10	0.1–0.4 µg/mL hypericin
[32]	Troichenko et al. (2021)	10 (UV) 3 (Laser)	0.1% riboflavin 0.1% methylene blue
[33]	Sakita et al. (2019)	120	0.25–32.0 µM P123-Hyp
[34]	Sato et al. (2022)	Not specified	Hypericin in lipid carriers (specific concentration unspecified)
[35]	Tunccan et al. (2018)	4320	0.1% riboflavin 25 μg/mL methylene blue 0.1% rose bengal
[36]	Rezusta et al. (2012)	Not specified	0.625–320 µM hypericin
[37]	Morelato et al. (2022)	Not specified	0.1% riboflavin 0.1% methylene blue
[38]	Paz-Cristobal et al. (2014)	1–60	0.625–40 µM hypericin 1.25–2.5 µM DMMB
[39]	Sato et al. (2023)		
[40]	Kashiwabuchi et al. (2013)	30	Riboflavin, UV-A (not quantified)
[41]	Sousa et al. (2019)	10 in PBS, 30 in plasma/whole blood	5.0 µM Tri-Py(+)-Me 10 µM FORM

DMMB—Dimethyl Methylene Blue, P123-Hyp—Hypericin Encapsulated in P123 Micelles, Tri-Py(+)-Me—Tri-Pyridyl Methyl, UV—Ultraviolet Radiotaion.

## Data Availability

Data are contained within the article.
